# Creative interventions: increasing enrollment, serving students & community, reaffirming gerontology education

**DOI:** 10.1093/geroni/igaa057.1754

**Published:** 2020-12-16

**Authors:** Jan Abushakrah, Mike Faber

## Abstract

The session will focus on innovative ways to increase student enrollment, better meet the needs of students and the communities served, and raise awareness of the crucial role of gerontology education in addressing the needs of a rapidly aging society. A panel of Community College and University gerontology professionals will share the innovative ways that they are working to address the three focus areas of this symposium. We will also include opportunities for discussion with participants about their experiences with and ideas for addressing these issues. Community College Interest Group Sponsored Symposium.

